# Exploiting uniqueness: seed-chain-extend alignment on elastic founder graphs

**DOI:** 10.1093/bioinformatics/btaf225

**Published:** 2025-07-15

**Authors:** Nicola Rizzo, Manuel Cáceres, Veli Mäkinen

**Affiliations:** Department of Computer Science, University of Helsinki, 00014 Helsinki, Finland; Department of Computer Science, Aalto University, 00076 Espoo, Finland; Department of Computer Science, University of Helsinki, 00014 Helsinki, Finland

## Abstract

**Summary:**

Sequence-to-graph alignment is a central challenge of computational pangenomics. To overcome the theoretical hardness of the problem, state-of-the-art tools use *seed-and-extend* or *seed-chain-extend* heuristics to alignment. We implement a complete seed-chain-extend alignment workflow based on *indexable elastic founder graphs* (iEFGs) that support linear-time exact searches unlike general graphs. We show how to construct iEFGs, find high-quality seeds, chain, and extend them at the scale of a telomere-to-telomere assembled human chromosome.

**Availability and implementation:**

Our sequence-to-graph alignment tool and the scripts to replicate our experiments are available in https://github.com/algbio/SRFAligner.

## 1 Introduction

The aim of computational pangenomics is to incorporate observed genetic variants into the analysis of sequencing data (Computational Pan-Genomics Consortium 2018). This is to overcome reference bias of a unique consensus genome (Garrison *et al.* 2018). To tackle the challenges involved, there exist two semantically different approaches under inspection: string collection-based and graph-based pangenomes. The former builds on compressed representations of a set of haplotype sequences (Mäkinen *et al.* 2010, Gagie *et al.* 2020, Chen *et al.* 2021, Rossi *et al.* 2022) and the latter explicitly compacts the shared sequence fragments into graph nodes, so that paths through the graph encode the haplotypes (Garrison *et al.* 2018, Eggertsson *et al.* 2019, Kim *et al.* 2019, Sirén *et al.* 2021, Baaijens *et al.* 2022, Chandra *et al.* 2024). When compared to the use of a single reference sequence, both approaches need further considerations to be useful in downstream analyses. In string collections, the shared content between haplotypes is compacted without alignment information, so e.g. a substring match between a sequencing read and the set of haplotypes can have many occurrence locations, but only a few of them represent genetically different loci. In pangenome graphs, haplotype information can be seen as a complex tailored compression scheme for the underlying set of genomes; achieving both the high compressibility and fast queries of string collection-based approaches is challenging.

Currently, there exist a diverse selection of graph-based pangenome representations being actively used in downstream applications with different tradeoffs (Andreace *et al.* 2023): tool vg (Garrison *et al.* 2018) builds a variation graph starting from a reference and a variant call file (VCF); de Bruijn Graphs collapse all occurrences of a *k*-mer (string of a fixed length *k*) into a single node (Eggertsson *et al.* 2019); minigraph (Li *et al.* 2020) and minichain (Chandra and Jain 2023b) perform incremental graph generation considering only variations of 50bps (base pairs) or more; minigraph-cactus (Hickey *et al.* 2024) and the pggb pipeline (Garrison *et al.* 2024) capture complex structural variations with whole-genome alignment and all-to-all pairwise alignment techniques, respectively.

**Figure 1.**
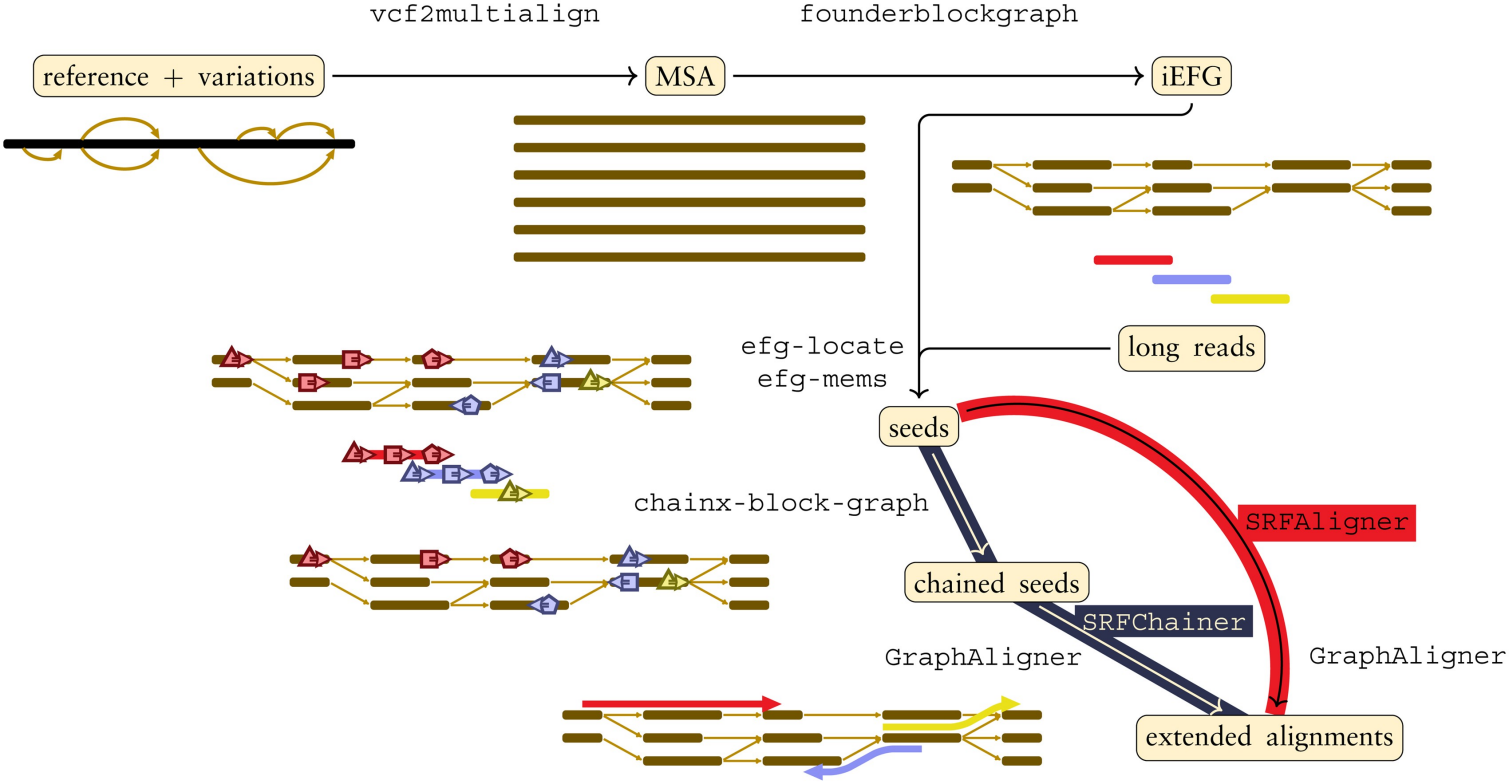
Workflow for the construction of iEFGs and the alignment of long reads: the variations (in VCF format) implicitly represent a graph that vcf2multialign (Norri *et al.* 2021) converts to a multiple sequence alignment (FASTA), which is then segmented into an iEFG (GFA). Then, the alignment (GAF) is performed by finding seeds between the reads or their reverse complement and the graph (GAF), optionally finding the optimal chains per strand, and passing the seeds to GraphAligner (Rautiainen and Marschall 2020).

*Sequence-to-graph* alignment has thus become a critical operation. There are haplotype-aware approaches for short read alignment (Sirén *et al.* 2021, Chandra *et al.* 2024) as well as speed-ups to the quadratic exact alignment algorithms (Jain *et al.* 2019, Darby *et al.* 2020, Marco-Sola *et al.* 2021, Ivanov *et al.* 2022, Zhang *et al.* 2022), that have potential for becoming practical also in long read alignment. As of now, to scale to massive dataset of long reads, state-of-the-art aligners adopt a heuristic *seed-and-extend* strategy [GraphAligner (Rautiainen and Marschall 2020)] sometimes leveraged by the more principled chaining technique into the *seed-chain-extend* approach [GraphChainer (Ma *et al.* 2023), minigraph (Li *et al.* 2020) and minichain (Chandra and Jain 2023b)]. These methods are illustrated in bottom-left of Fig. 1: seeding refers to the finding of short (exact) matches to anchor the alignments, chaining to the selection of a subset of anchors to form a skeleton of the alignment, and extension for filling the gaps to form a full alignment. These methods risk missing optimal alignments to satisfy the demands of large genomic databases. For scalability, such risk may be unavoidable, as it is known that a subquadratic-time algorithm for alignment is unlikely to exist (Backurs and Indyk 2018, Jain *et al.* 2020) and furthermore, the more fundamental exact matching problem on graphs is similarly difficult (Equi *et al.* 2023a,b) even when considering limited-topology objects such as elastic degenerate strings (EDSes) (Gibney 2020) or elastic founder graphs (EFGs) (Equi *et al.* 2023c).

In this paper, we demonstrate the practical performance of *indexable* (a.k.a. semi-repeat-free) EFGs (iEFGs) (Equi *et al.* 2023c) at the scale of telomere-to-telomere assembled human chromosome level. As a principled approach to graph-based pangenomics, iEFGs are built from a set of aligned sequences that are only allowed to recombine after sharing a unique genomic fragment. Moreover, iEFGs leverage the power of collection-based techniques to support fast exact pattern matching queries on the graph (Rizzo *et al.* 2024). We exploit these properties to obtain a scalable full alignment pipeline for iEFGs depicted in Fig. 1.

In more detail, our main contributions are:

- a practical fast exact matching solution on iEFGs as tool efg-locate;
- a new greedy seeding technique exploiting the uniqueness of iEFGs, finding *semi-repeat-free* (srf) seeds;
- an extension of the ChainX (Jain *et al.* 2022a) algorithm on sequences to iEFGs as tool ChainX-block-graph;
- a complete sequence-to-graph workflow, by feeding to GraphAligner (Rautiainen and Marschall 2020) the srf seeds, after optionally chaining them.

### 1.1 Structure of the paper and summary of our results

In the next section, we introduce the basic concepts around iEFGs. Then, in Section 3, we introduce srf-based seeds and a greedy seeding algorithm to find them. We continue in Section 4 by tailoring a ChainX-based (Jain *et al.* 2022a) chaining algorithm to our seeds on the EDS relaxation of iEFGs, obtaining an algorithm running in O(n·OPT+n log n) average-case time, where *n* is the number of seeds and OPT is the cost of the chain. In Section 5, we compare our approaches with state-of-the-art aligners and show that our workflow achieves competitive accuracy while being 4.5 times faster than GraphAligner (Rautiainen and Marschall 2020) on the same input graph and 1.5 times faster than GraphAligner on vg graphs.

Our final product is a toolset consisting of SRFAligner and SRFChainer that implement the two alternative pipelines of the workflow from Fig. 1. Its components are standalone programs that can be used independently, since we believe that each technique of our pipeline is of independent interest. We provide thorough experiments and full details in our Supplement. This main paper focuses on the overall workflow.

## 2 Preliminaries

Given positive integers x≤y, we denote range {x,x+1,…,y} as [x..y]. Given a finite alphabet Σ, we say that $Q=Q[1]Q[2]\cdots Q[n]\in \Sigma^{n}$ is a string of length *n* over Σ, and also that |Q|=n. Then, we define $\Sigma^{*}$ as the set of all strings over Σ and $\Sigma^{+}=\Sigma \setminus \{\varepsilon \}$, where ε is the string of length 0. We also define Q[i..j] as the concatenation Q[i]Q[i+1]⋯Q[j] of the symbols of *Q* from the *i*th to the *j*th. String *P* is then a *substring* of *Q* if P=Q[i..j] for some i,j∈[1..|Q|]: if P≠Q then we say that *P* is *proper*; if i=1 then *P* is a *prefix* of *Q* and we also write Q[..j]; if j=|Q| it is a *suffix* and we also write Q[i..]. Further, we say that *i* is an *occurrence* of *P* in *Q*, and if P[x..y]=Q[a..b] then ([x..y],[a..b]) is an *(exact match) anchor* between *P* and *Q*.

**Figure 2.**
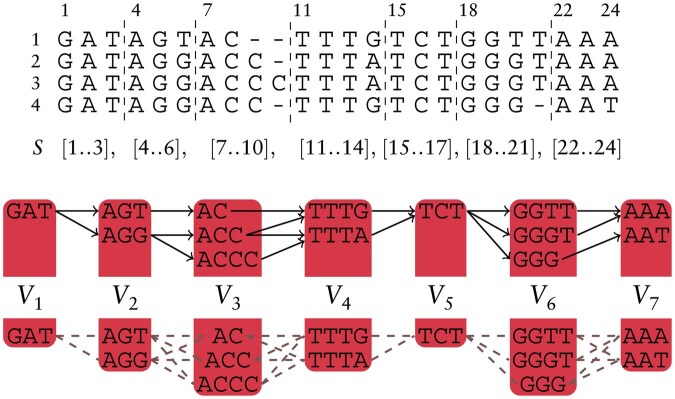
An example of an MSA[1..4,1..24], segmentation *S*, and iEFG G=(V,E,ℓ) induced by *S* of height H(G) equal to 3 and maximum node length L(G) equal to 4. Below, the relaxation of the iEFG to an elastic degenerate string G=(V,ℓ) allowing all possible edges between consecutive blocks.

In this paper, we deal with graphs built from the segmentation of multiple sequence alignments, represented as a matrix $\mathrm{MSA}[1..m,1..n]\in {\left(\Sigma \cup \{-\}\right)}^{m\times n}$ representing *m* sequences (the rows MSA[i,1..n] for i∈[1..m]) aligned into *n* positions (the columns). Alphabet Σ is expanded with the special *gap character* -∉Σ representing insertions and deletions: given an aligned sequence MSA[i,1..n] we can retrieve the original sequence spell(MSA[i,1..n]) by removing the gaps. We define a *segmentation S* of MSA[1..m,1..n] as a partition of columns [1..n] in *b* segments $\left[1=x_{1}..y_{1}],\ldots ,[x_{b}..y_{b}=n\right]$ such that $y_{i}=x_{i+1}-1$ for i∈[1..b−1]. We consider the following graph objects, see also Fig. 2.

Definition 1 [elastic founder graph (Equi *et al.* 2023c)].

Let $\mathrm{MSA}[1..m,1..n]\in {\left(\Sigma \cup \{-\}\right)}^{m\times n}$ be a multiple sequence alignment. Given segmentation $S=[x_{1}..y_{1}]$, …, $\left[x_{b}..y_{b}\right]$, the *elastic founder graph* (EFG) *induced by*  S is a vertex-labeled graph G=(V,E,ℓ), where $\ell :V\to \Sigma^{+}$ assigns a nonempty *label* to every node, and the set of nodes V is partitioned into blocks $V_{1}$, …, $V_{b}$ such that $\left|\{\ell (v):v\in V_{k}\}|=|V_{k}\right|$ (unique strings in a block) and for all k∈[1..b] we have that

$$\{\ell (v):v\in V_{k}\}=\{\mathrm{spell}(\mathrm{MSA}[i,x_{k}..y_{k}])\;|\;1\leq i\leq m\}.$$

Moreover, it holds that (v,w)∈E if and only if there exists k∈[1..b−1] and i∈[1..m] such that $v\in V_{k}$, $w\in V_{k+1}$, and $\mathrm{spell}(\mathrm{MSA}[i,x_{k}..y_{k+1}])=\ell (v)\ell (w)$.

If $E=\overset{b-1}{\underset{k=1}{\cup }}\left(V_{k}\times V_{k+1}\right)$, i.e. all nodes in $V_{k}$ are connected to all nodes in $V_{k+1}$ for each b∈[1..b], then the EFG is an elastic degenerate string (EDS) (Grossi *et al.* 2017), and we can omit edge set *E*. Moreover, given an EFG, we define its EDS relaxation as the corresponding EDS over $V_{1},\ldots ,V_{b}$.

Given an elastic founder graph G=(V,E,ℓ), for each node v∈V we denote as ‖*v*‖ the node label length |ℓ(v)|. We define height $H(G)=\max_{k=1}^{b}|V_{k}|$ as the maximum number of nodes in a block, and $L(G)=\max_{v\in V}\|v\|$ as its maximum node label length. We can extend ℓ to label paths in the graph: given a $u_{1}u_{k}$-path $P=u_{1}\cdots u_{k}$, with $(u_{i},u_{i+1})\in E$ for i∈[1..k−1], we define $\ell (P)=\ell (u_{1})\cdots \ell (u_{k})$; for an edge (u,v)∈E, we call ℓ(uv) its *edge label*. Then, we say that some string $Q\in \Sigma^{+}$  *occurs* in *G* if there exists path *P* in *G* such that *Q* is a substring of ℓ(P). More specifically, we indicate with triple $\left(i,u_{1}\cdots u_{k},j\right)$ the *subpath* of *P spelling Q* starting from position $i\in [1..\|u_{1}\|]$ in $u_{1}$ and ending in position $j\in [1..\|u_{k}\|]$ in $u_{k}$, i.e. $Q=\ell (u_{1})[i..]\ell (u_{2}\cdots u_{k-1})\ell (u_{k})[..j]$. The *length* of subpath $\left(i,u_{1}\cdots u_{k},j\right)$ is then |*Q*|. We say that *Q* occurs *starting from the beginning of* $u_{1}$ if i=1; *Q* occurs *starting inside* $u_{1}$ if $i\in [2..\|u_{1}\|]$. Moreover, if substring Q[x..y] is spelled by subpath $\left(i,P=u_{1}\cdots u_{k},j\right)$, we say that pair ([x..y],(i,P,j)) is an *(exact match) anchor* between *Q* and *G*, and if k=1 we say that it is a *node anchor*.

Unfortunately, EFGs are still hard to index for subquadratic-time pattern matching (Equi *et al.* 2023c), and an additional uniqueness property such as the following one is required.

Definition 2 [semi-repeat-free indexability property (Equi *et al.* 2023c)].

An EFG G=(V,E,ℓ) is *semi-repeat-free* or *indexable* (denoted as iEFG) if each subpath $\left(i,u_{1}\cdots u_{p},j\right)$ spelling ℓ(v) for $v\in V_{k}$ is such that i=1 and $u_{1}\in V_{k}$, i.e. ℓ(v) only occurs from the beginning of nodes in block $V_{k}$.

Lemma 1 [semi-repeat-free uniqueness properties (Equi *et al.* 2023c)]. Given an iEFG G=(V,E,ℓ):

1. any two distinct nodes u≠v, u,v∈V, cannot have the same node label, i.e. ℓ(u)≠ℓ(v);
2. for any u,v∈V, while ℓ(u) can be prefix of ℓ(v), ℓ(u) cannot be a proper suffix of ℓ(v);
3. all occurrences of ℓ(uv) in G with u,v∈V start from the beginning of *u*.

Although the semi-repeat-free property of the iEFG was developed solely to enable fast construction and fast queries (Rizzo *et al.* 2024), it has an interesting analogy to biological recombination: in both cases, sequence similarity is a prerequisite of recombination. In particular, the iEFG requires a unique string between two aligned haplotypes (not appearing elsewhere) before it allows these haplotypes to recombine. We hypothesize that this feature helps to reduce spurious recombinations (those not feasible biologically) when compared to other pangenome graphs.

To index iEFGs and EDSes, we use the well-known machinery of text indexing and bit vectors behind the FM-index (Ferragina and Manzini 2000). For a bit array $B[1\ldots n]\in {\left\{0,1\right\}}^{n}$, we use *rank* and *select* queries over *B*: given a position i∈[1..n], rank answers the number of ones in B[1..i], whereas given a rank position j∈[1..rank(n,B)], selects return the position of the *j*th one in *B*. The bit vectors used can be preprocessed to answer these queries in constant time (Navarro 2016). Besides, we preprocess a text *T* for *backward pattern matching* based on the *suffix array*: we consider the suffixes T[i..n+1] of T$ in their lexicographical order, with $∉Σ a special terminator character; we can search for the occurrences of pattern $Q\in \Sigma^{+}$ in *T* by iteratively considering the range [ℓ..r] in the list of sorted suffixes corresponding to suffix Q[i..]; importantly, this range corresponds to all occurrences of Q[i..] in *T*. The main primitive of backward search is then operation leftExtend(T,[ℓ..r],c), that given c∈Σ and interval [ℓ..r] corresponding to string *Q* returns the interval corresponding to string c·Q. This operation can be supported in constant time (Belazzougui and Navarro 2014).

## 3 Exact pattern matching and unique seeds

Indexable EFGs admit linear-time exact pattern matching by indexing (a variation of) the following string spelled by the concatenation of edge labels (Rizzo *et al.* 2024):


(1) $$T_{\text{edges}}=\#\cdot \prod_{(u,v)\in E}\ell (uv)\#,$$

that is $T_{\text{edges}}$ is the concatenation of the edge labels separated by a special # character and by construction each position $j\in [1..|T_{\text{edges}}|]$ such that $T_{\text{edges}}[j]\neq \#$ corresponds to position *i* in ℓ(uv) for exactly one edge (u,v)∈E and vice versa. During matching, when a pattern spans more than two nodes, special attention must be given to the last few blocks matching the string: in (Rizzo *et al.* 2024, Sections 3 and 6), this is solved by complex algorithms requiring several advanced data structures. This algorithm has not been implemented. We give a simplified solution that has a slower worst-case running time of $O(|Q|+\min{\left(|Q|,L(G)\right)}^{2}+H{\left(G\right)}^{2})$ but is amenable to a practical implementation that we later extend to a new seeding technique. In particular, we require only constant-time support for the two following operations:

-
$\text{leftExtend}(T_{\text{edges}},[\ell ..r],c)$ , given c∈Σ and the suffix array interval [ℓ..r] corresponding to string *S* for $T_{\text{edges}}\cdot \$$, returns the suffix array interval corresponding to c·S if it occurs in $T_{\text{edges}}$, otherwise the empty interval;
-
$\text{edgeLocate}(T_{\text{edges}},\ell )$ , given a suffix array index ℓ for $T_{\text{edges}}\cdot \$$ corresponding to a suffix of ℓ(uv), (u,v)∈E, starting at position *i* in such suffix, returns triple (u,v,i).

**Figure 3.**
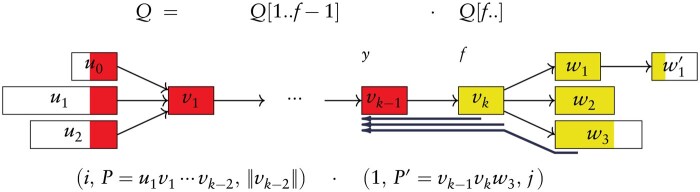
Illustration of the occurrences of a long pattern in an iEFG and of the pattern matching executed by Algorithm 1: the first search finds position *f*, and corresponds to the red nodes; the second search, corresponding to the yellow nodes, finds an occurrence of Q[1..f−1] (omitting its last node $v_{k}-1$) and position *y*; the final step, corresponding to the dark blue arrows, connects the two searches and finds a compatible occurrence of Q[y..].

The exact pattern matching algorithm is outlined in Algorithm 1 and its main strategy is visualized in Fig. 3: a first search (subroutine F) finds the longest suffix Q[f..] such that Q[f..] is a prefix of some edge ℓ(uv), (u,v)∈E; a second search (subroutine S) finds an occurrence of Q[1..f−1] in *G*, on the condition that this occurrence of Q[1..f−1] must end at a node boundary; a final search (subroutine C) connects Q[1..f−1] to Q[f..] by testing for a matching vertex between the occurrences of $Q[1..f^{\prime }]$ and those of $Q[f^{\prime }+1..]$, for $f^{\prime }\in [f..|Q|]$. We refer the reader to the Supplement for the description of the subroutines and for the proof that these searches correctly answer exact pattern matching.

Algorithm 1 High-level overview of exact pattern matching of a pattern *Q* in iEFG *G* in $O(|Q|+\min{\left(|Q|,L(G)\right)}^{2}+H{\left(G\right)}^{2})$ time after $O(|T_{\text{edges}}|)$-time preprocessing. A full description of the subroutines is given in the Supplement.

**Input:** An EFG G=(V,E,ℓ) and query string $Q\in \Sigma^{+}$

**Output:** Match (i,P,j) of *Q* in *G* if it exists, otherwise **false**

1: Compute $T_{\text{edges}}=\#\cdot \prod_{(u,v)\in E}\ell (uv)\#$

2: Preprocess $T_{\text{edges}}$ for backwards pattern matching and edge locate queries

3: Execute subroutine F for the first search and store result in *f*

4: Execute subroutine S for the subsequent searches and store results in *P*, *i*, and *y*

5: Execute subroutine C for the connection and store results in $P^{\prime }$ and *j*

6: **return**  $\left(i,P\cdot P^{\prime },j\right)$

In order to exploit the uniqueness properties of iEFGs as a seeding technique, we formalize the following type of seed.

Definition 3 (Semi-repeat-free seed). *An anchor* $\left([x..y],(i,P=u_{1}\cdots u_{k},j)\right)$  *between iEFG*  G=(V,E,ℓ)  *and pattern* $Q\in \Sigma^{+}$  *is a semi-repeat-free seed if subpath* (i,P,j)  *spans at least a full node, defined as follows:* k>2*, or if* k=1  *then* i=1  *and* $j=\|u_{k}\|$*, or* k=2  *and at least one of conditions* i=1  *or* $j=\|u_{k}\|$  *hold.*

Semi-repeat seeds can be found easily by feeding the queries to the Aho-Corasick automaton of the node labels. It turns out that for efficient chaining we need an additional property for seeds not to overlap in the query. Hence, we instead use a greedy approach to find a subset of semi-repeat-free seeds with this property. In our algorithm, we apply exact pattern matching iteratively as follows:

1. We use Algorithm 1 to find the longest suffix Q[y..] matching in *G*;
2. We report one exact match (Q[y..],(i,P,j)) only if it spans at least one full node;
3. We restart the search (step 1) on the remaining prefix Q[1..y−1] unless y−1=0.

We expect this greedy subset of semi-repeat-free seeds to be of high quality in practice for the following reasoning. Consider the case $Q[x..y]=\ell (u_{1}\cdots u_{k})$, with k≥4 and $u_{1}\cdots u_{k}$ a path in *G*. By the third property in Lemma 1, all occurrences of $\ell (u_{k-1}u_{k})$ in *G* start from the beggining of $u_{k-1}$, thus our greedy approach will either report the entire match Q[x..y] or stop inside $u_{k-1}$; in the latter case, the search will anyway restart and find the semi-repeat-free seed corresponding to $\ell (u_{1}\cdots u_{k-3})$.

We implemented the above solutions to pattern matching and seeding in our tool efg-locate.

## 4 Chaining on elastic founder graphs

We show how to chain a set of exact match node anchors between a read and an iEFG by adapting the sequence-to-sequence co-linear chaining formulation introduced by Jain *et al.* (2022a). We dedicate this section to explain how the work of Jain *et al.* (2022a) works in the sequence-to-sequence case, and our specific modifications to adapt this solution for the sequence-to-iEFG case. First, we define the chaining problem between *two sequences*.

Definition 4 [co-linear chain (Jain *et al.* 2022a)]. *Given strings* $T_{1},T_{2}\in \Sigma^{+}$*, let* $A_{p}=([x_{p}..y_{p}],[a_{p}..b_{p}])$, $A_{q}=([x_{q}..y_{q}],[a_{q}..b_{q}])$  *be two exact match anchors between* $T_{1}$  *and* $T_{2}$*. Interval [x..y] precedes interval* $\left[x^{\prime }..y^{\prime }\right]$  *if* $x\leq x^{\prime }$  *and* $y\leq y^{\prime }$*. We say that* $A_{p}$  *precedes* $A_{q}$  *if both* $\left[x_{p}..y_{p}\right]$  *precedes* $\left[x_{q}..y_{q}\right]$  *and* $\left[a_{p}..b_{p}\right]$  *precedes* $\left[a_{q}..b_{q}\right]$*. In this case*, $A_{p}$  *and* $A_{q}$  *are said to be co-linear. A sequence of anchors* $A_{1},\ldots ,A_{c}$  *is called a (co-linear) chain if* $A_{p}$  *precedes* $A_{p+1}$  *for all* p∈[1…c−1]*. The cost of a chain* $A_{1},\ldots ,A_{c}$  *is* $\sum_{p=1}^{c-1}\mathrm{connect}(A_{p},A_{p+1})$*, where* connect  *is defined as* $\mathrm{connect}(A_{p},A_{p+1})=g(A_{p},A_{p+1})+o(A_{p},A_{p+1})$  *with*
 

$$g(A_{p},A_{p+1})=\max(0,x_{p+1}-y_{p}-1,a_{p+1}-b_{p}-1),\;\;\;\;\;\;\;\;\;\;\;\;\;\;\;\;\;\;\;\;\;\;\;\;\;\;\;\;\;\;\;\;\;\;\;\;(\text{gapcost})o(A_{p},A_{p+1})=|\max(0,y_{p}-x_{p+1}+1)-\;\max(0,b_{p}-a_{p+1}+1)|.\;\;\;\;\;\;\;\;\;\;\;\;\;(\text{overlapcost})$$

Note that the cost of a chain is the total sum of the connect values of anchors consecutive in the chain. In (Jain *et al.* 2022a), the authors prove that the minimum cost of a chain is equal to the edit distance supported by the anchors [This specialized version of edit distance is called anchored edit distance in the paper. In particular, the equivalence holds after adding two special initial and final anchors. By changing the connect function for these two special anchors, the minimum cost of a chain can become the semi-global (anchored) edit distance between the string and the graph, which is the objective function used for long reads.], thus establishing an elegant connection between edit distance and co-linear chaining. They also show that such a minimum cost chain can be computed by a simple dynamic program in $O(n^{2})$ time, where *n* is the number of anchors: for each anchor $A_{q}$, compute the minimum cost C[q] of a chain ending with anchor $A_{q}$, by taking the minimum of $C[p]+\mathrm{connect}(A_{p},A_{q})$ over all anchors $A_{p}$ preceeding $A_{q}$. Moreover, they solve this problem in $O(n{\;\text{log}\;}^{4}n)$ time using 4D data structures and they propose a simpler and practical algorithm that restricts the dynamic programming search to anchors at distance at most *B* in one of the strings, a parameter which is initially set to the initial guess $B_{1}$. Then, since the optimal solution might contain consecutive anchors at distance more than $B_{1}$, at each iteration the algorithm updates *B* to B·α, where α>1 is a given ramp-up parameter. Even though this approach runs in $O(n^{2})$ time in the worst case, Jain *et al.* show that under a uniform and sparse distribution of anchors the algorithm runs in $O(n\cdot B_{\text{max}}+n\;\text{log}\;n)$ time, where $B_{\text{max}}$ is the maximum value of *B* considered. In the conference version of the work (Jain *et al.* 2022b), it was initially claimed that the algorithm finds the optimal solution and the solution quality was verified experimentally. Unfortunately, optimality is not always guaranteed *see*  *Jain et al.* (2022a, Section 5), but the experimental results hold nonetheless. This practical solution was implemented into the tool ChainX [Available at https://github.com/at-cg/ChainX.].

Here, we adapt ChainX’s algorithm for sequence-to-EDS chaining. In our workflow, we apply this exact solution as a heuristic for co-linear chaining on iEFGs, given their similar structure: conceptually, we just add all possible edges between adjacent iEFG blocks to obtain an EDS; we also split each seed spanning multiple nodes into multiple seeds spanning only one node. Even though the EDS chain might not be a valid chain in the iEFG, we can still use the chained anchors to obtain high-quality seeds guiding the extension phase. Moreover, this heuristic approach is simpler and faster compared to chaining the original seeds on the iEFG. Additionally, we show that by a small modification of the algorithm by Jain *et al.* we recover the optimality claim for some of the anchors used in our experiments: instead of checking the distance between the starting positions of anchors, we check for the gap between them.

To adapt ChainX, we show how to perform the following two operations in constant time: computing the connect function between anchors, and deciding whether an anchor precedes another in the EDS.

**Figure 4.**
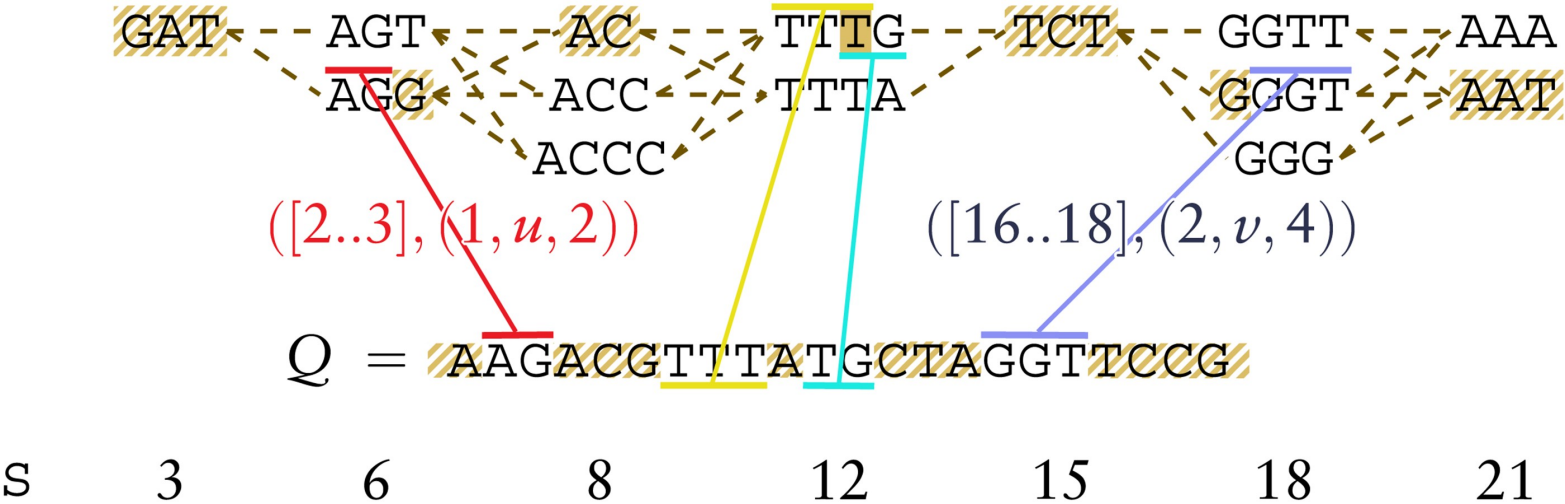
An EDS G=(V,ℓ) partitioned into blocks $V_{1}$, …, $V_{7}$ (degenerate letters in EDS notation), a pattern *Q*, and a chain of co-linear node anchors between *Q* and *G*. The gaps between adjacent anchors are marked with stripes, whereas overlaps are marked with a solid background. The cost of connecting consecutive anchors in the chain, defined as the maximum gap plus the absolute overlap difference, can be computed in constant time by preprocessing *G* for the constant-time computation of node block indices and distance between two EDS nodes.

Definition 5 [co-linear chain on a graph (Chandra and Jain 2023a)]. *Given* $Q\in \Sigma^{+}$  *and labeled graph* G=(V,E,ℓ)*, let* $A_{p}=([x_{p}..y_{p}],(i_{p},u_{p},j_{p}))$  *and* $A_{q}=([x_{q}..y_{q}],(i_{q},u_{q},j_{q}))$  *be exact match anchors between Q and G. Then*, $A_{p}$  *precedes* $A_{q}$  *if* $\left[x_{p}..y_{p}\right]$  *precedes* $\left[x_{q}..y_{q}\right]$  *and:* $u_{p}\neq u_{q}$  *and there is a* $u_{p}u_{q}$*-path, or* $u_{p}=u_{q}$  *and* $\left[i_{p}..j_{p}\right]$  *precedes* $\left[i_{q}..j_{q}\right]$*. The overlap in the graph is zero if* $u_{p}\neq u_{p+1}$  *and as before otherwise. The gap in the graph is defined as before if* $u_{p}=u_{p+1}$  *and equal to the shortest sequence length of a* $u_{p}u_{p+1}$*-path minus* $\left(j_{p}+||u_{p+1}||-i_{p+1}+1\right)$  *otherwise. See Fig. 4*.

First, to decide if $A_{p}=([x_{p}..y_{p}],(i_{p},u_{p},j_{p}))$ precedes $A_{q}=([x_{q}..y_{q}],(i_{q},u_{q},j_{q}))$, we precompute a table B such that $B[u]=V_{i}$ is the block of $u\in V_{i}$ for each i∈[1..b]. For $A_{p}$ to precede $A_{q}$, $\left[x_{p}..y_{p}\right]$ must precede $\left[x_{q}..y_{q}\right]$. Moreover, if $u_{p}=u_{q}$, then $\left[i_{p}..j_{p}\right]$ must precede $\left[i_{q}..j_{q}\right]$, and if $u_{p}\neq u_{q}$, then $B[u_{p}]<B[u_{q}]$ must hold. In any other case $A_{p}$ does not precede $A_{q}$. For the connect function, we re-use the overlap function of ChainX (as overlaps in the EDS can only be computed when $u_{p}=u_{q}$) and we compute the gap function as follows. Again, if $u_{p}=u_{q}$ then we reuse the gap function from ChainX. Otherwise, if $B[u_{p}]<B[u_{q}]$ we define the gap (in the EDS) as $\left\|u_{p}\|-j_{p}+\sum_{i=B[u_{p}]+1}^{B[u_{q}]-1}\min_{u\in V_{i}}\|u\|+(i_{q}-1\right)$, i.e. the distance from $j_{p}$ to the end of $\ell (u_{p})$, plus the minimum label length of each block inbetween $u_{p}$ and $u_{p}$, plus the distance from the beginning of $\ell (u_{q})$ until $i_{q}$ (corresponding graph distance when the EDS is interpreted as a graph). Note that this value can be computed in constant time by precomputing values S[1..b] such that S$\left[i]=\sum_{j=1}^{i}\min_{v\in V_{j}}\|v\right\|$. See Fig. 4 for an example. See the Supplement for the pseudocode summarizing our adaptation of ChainX and its proof of correctness.

The semi-repeat-free seeds and their variations used in our experiments do not overlap in *Q* with other co-linear anchors. As such, every minimum cost chain is made of non-overlapping anchors in *Q*; we show in the Supplement that this guarantees correctness of the approach on our datasets. Moreover, for these classes of inputs, we recover the average-case running time guarantee, assuming a uniform anchor distribution and n≤|Q|. See the Supplement for the proof of the following result.

Theorem 2. *Let* $Q\in \Sigma^{+}$  *be a pattern and* G=(V,ℓ)  *an EDS preprocessed for constant-time precedence and connect queries. Given n anchors between Q and G, we can find an optimal chain of cost* OPT  *in* O(n·OPT+n log n)  *average-case time, assuming that* n≤|Q|*, the anchors do not overlap in the query, and the anchor endpoints are uniformly distributed.*

We implemented co-linear chaining on the EDS relaxation of an iEFG in tool Chainx-block-graph. The tool supports global and semi-global chaining and accepts the following user-defined parameters: the initial guess $B_{1}$ for the optimal cost and the ramp-up factor α. Additionally, a constant initial guess can be replaced with the guess β·(|Q|−c), where *c* is the coverage of read *Q* by the input seeds.

## 5 Experiments

We built chromosome iEFGs from the recent T2T-CHM13 reference genome and the variation from the 1000 Genomes Project (1KGP) recalled and phased on this reference (Nurk *et al.* 2022, Lalli 2023, Rhie *et al.* 2023). For our experiments, we use the chromosome 22 iEFG, denoted as chr22-iEFG. We do not expect results to change relatively with larger chromosomes unless there are longer gap regions, and this choice enables more extensive testing of different parameter combinations.

The machine used in the experiments runs on an Intel(R) Xeon(R) CPU E7-4830 v3 @ 2.10 GHz and 1.5 TB of RAM. SSD storage space is used in all experiments except in the graph construction experiments, where a large amount of disk space was needed and HDD storage space was used. Unless stated otherwise, each tool is executed using 64 threads, and the aligners do not use additional disk space nor reuse indexes for the graph or the reads.

### 5.1 Pangenome graph construction

The various engineering choices required to make this feasible on top of the earlier theoretical construction algorithms (Rizzo *et al.* 2024) are detailed in the Supplement. The Supplement also contains a thorough study of the uniqueness of a human pangenome, as well as a comparison to graphs built with the vg toolkit (Garrison *et al.* 2018), summarized in Table 1. We tested two vg graphs, one built from the same VCF file, chr22-vg-VCF, and another built from the corresponding MSA obtained with vcf2multialign (Norri *et al.* 2021), chr22-vg-MSA. Additionally, we created chr22-vg-VCF-u and chr22-vg-MSA-u, obtained after merging nonbranching paths of chr22-vg-VCF-u and chr22-vg-MSA-u, respectively. We use this latter pair of graphs in our experiments [We did this as some tested tools did not find any alignments on chr22-vg-VCF and chr22-vg-MSA.].

**Table 1.** Features of the chromosome 22 graphs generated from the T2T-CHM13 reference and the 1KGP phased variations.^a^

Graph	Construction time	Construction memory
chr22-vg-VCF	11 m	569 MB
chr22-vg-MSA	24 h	400 GB
chr22-iEFG	17 h	595 GB

Graph	Nodes	Edges	Bases	N50	Longest node	*H*	Width
chr22-vg-VCF	4 839 050	6 408 323	53 073 716	32	32		28
chr22-vg-VCF-u	3 758 151	5 327 424	53 073 716	75	2 102 234		28
chr22-vg-MSA	4 787 254	6 145 861	52 782 038	32	32		9
chr22-vg-MSA-u	3 702 599	5 061 206	52 782 038	77	2 102 234		9
chr22-iEFG	3 639 676	5 036 647	88 930 533	26	356 478	1313	1313

Graph	Branching nodes	Choices	Branching factor	Paths
chr22-vg-VCF	1 423 765	2 993 039	1 307 293	5.19 × 10^401730^
chr22-vg-VCF-u	1 423 765	2 993 039	1 307 293	5.19 × 10^401730^
chr22-vg-MSA	1 323 783	2 682 391	1 321 412	2.37 × 10^381472^
chr22-vg-MSA-u	1 323 783	2 682 391	1 321 412	2.37 × 10^381472^
chr22-iEFG	855 577	2 252 549	831 519	2.15 × 10^265424^

a Height *H* is defined as the largest number of nodes in an iEFG block; width is the size of the smallest set of paths covering the graph nodes; choices is the cumulative degree of branching nodes; branching factor is the largest number of branching nodes encountered in any path; paths is the total number of maximal paths expressed in the graph, represented in scientific notation rounded to the second decimal.

For iEFGs, we have two construction strategies (a) and (b), that produce slightly different graphs. For strategy (a), we follow the theoretical approach (Rizzo *et al.* 2024) in computing values f(j) such that columns [j..f(j)] of the MSA induce a semi-repeat free block in iEFG (see Supplement for details). To improve the running time, we implemented strategy (b) to approximate the f(j) values by partitioning the rows of the MSA into *k* disjoint sets, computing in parallel the *k* f(j) value approximations for each *j*, and picking the maximum of each. Then we applied the segmentation algorithm by Rizzo *et al.* (2024) on these approximate values. The resulting EFG is no longer guaranteed to be indexable, but it turns out that only some local adjustments (merging blocks that violate the indexability condition) are needed to make the graph indexable. We use the graph of strategy (b) in our read alignment experiments.

In all cases, graphs produced by vg contain several orders of magnitude more paths than iEFGs, showing that iEFGs effectively reduce the number of spurious recombinations.

### 5.2 Application and performance of exact search

An anticipated application for exact matching in a pangenome graph is the mapping of samples to subpopulations: Consider building pangenomes specific to populations in two geographic regions A and B. Given a set of reads from a new sample, compute the number of reads aligning exactly (with zero errors) in the two pangenomes. One could predict that the sample belongs to the subpopulation with higher number of exact hits. To make this application feasible, one needs a fast way to find the exact hits.

Indeed, one of the unique properties of iEFGs is their ability to theoretically support exact search on graphs as fast as many indexes support exact search on strings. We wanted to test how close its practical performance is from the highly engineered string indexes, i.e. if the application sketched above is feasible. For this we used the downsampled set of 338M reads from the ERR1025645 sequencing run (whole genome, first pair of paired-end reads) *used in*  *Norri et al.* 2021 from the Simons Genome Diversity Project (Mallick *et al.* 2016). Our tool efg-locate (on 16 threads) took 1 h 11 min for chr22-iEFG to locate exact occurrences of 7 067 538 reads (2.09% of total) or their reverse complement in chromosome 22. For comparison, bwa (on 16 threads) (Li and Durbin 2009) built on the same reference chromosome took 48 min to locate the exact occurrences of 6 833 767 reads (2.02% of total) or their reverse complement. Since the iEFG contains variations on top of the reference chromosome, the higher number of exact occurrences is anticipated. The small time difference indicates that applications building on exact search on graphs are fully feasible.

For comparison, we tried to repeat the same test with vg (Garrison *et al.* 2018) (reference + variants) and br-index (Arakawa *et al.* 2022) (MSA rows), but they timed-out (after 24 h they had not finished the runs).

### 5.3 Setup for alignment accuracy experiment

We measure the *accuracy* of the aligners as the numbers of correctly aligned reads divided by the total number of input reads. Sequence-to-graph aligners align each read to a subpath of the graph: we call such a path the *reported subpath* and the string spelled by this the *reported sequence*. If there are multiple reported alignments per read, we take the primary one (the first) and discard the secondary alignments (the rest) for GraphAligner-based tools, and for other tools we take the alignment with the longest reported subpath [We observed better accuracy with this method for other tools, as reported in (Ma *et al.* 2023)]. We define the correctness of an alignment with three different parameterized criteria introduced by Ma *et al.* (2023):

- (Path accuracy) Given a parameter 0<δ≤1 we say that an alignment is correct if the base-level overlap between the reported subpath and the true subpath is at least δ times the true subpath length. This is a generalization of the widely used 10% overlap criteria, i.e. δ=0.1 (Li 2018, Rautiainen and Marschall 2020, Chandra and Jain 2023a).
- (Truth edit distance accuracy) Given $0<\sigma_{\text{truth}}\leq 1$ we say that the alignment is correct if the Levenshtein distance between the reported sequence and true sequence is at most $\sigma_{\text{truth}}$ times the length of the true sequence.
- (Read edit distance accuracy) Given $0<\sigma_{\text{read}}\leq 1$ we say that the alignment is correct if the Levenshtein distance between the read and the reported sequence is at most $\sigma_{\text{read}}$ times the read length.

The ground truth is generated by choosing a random path through the graph in question and sampling long reads from it using BadRead simulator (Wick 2019). The simulator is run with its default error level of 5% under its Oxford Nanopore R10.4.1 model, target coverage of 30x, and target length of 15 000 bp.

Our final toolset consists of SRFAligner and SRFChainer which implement the two alternative full pipelines of the workflow depicted in Fig. 1, the former without chaining and the latter with chaining. In addition, they support parameterized mode -o X, that recovers the seeds corresponding to the X longest substrings of *Q* occurring in *G* found by the greedy seeding algorithm.

### 5.4 Alignment accuracy comparison

Table 2 shows the comparison of different modes of SRFAligner and SRFChainer to default GraphAligner (Rautiainen and Marschall 2020) as well as to minigraph (Li *et al.* 2020), minichain (Chandra and Jain 2023b), and GraphChainer (Ma *et al.* 2023). The column *seeds* is the number of seeds provided to the extension phase. The top part of the table shows the results when vg is used for building the graph, while the bottom part uses the iEFG. Note that the top and bottom parts use reads generated from the respective graphs, so the accuracy results are not directly comparable.

**Table 2.** Comparison of aligner performance and accuracy of simulated reads for the chromosome 22 graphs built from the T2T-CHM13 + 1KGP dataset.^a^

Graph	Aligner	Time (min)	Space (GB)	Seeds	Path accuracy		Edit distance accuracy	
					δ=0.1	δ=0.95	$\sigma_{\text{truth}}=0.1$	$\sigma_{\text{read}}=0.1$
chr22-vg-VCF-u	GraphAligner	9.66	**6.379**	2 463 181 632	**95.23**	91.11	96.10	88.00
	GraphChainer	68.00	31.148	4 926 363 264	93.71	*93.00	*97.02	*89.44
	minigraph	**6.42**	13.505	**1 441 813 071**	89.71	40.55	57.77	41.62
	minichain	33.68	38.260	**1 441 813 071**	54.23	23.54	28.91	22.24
chr22-iEFG	GraphAligner	27.83	10.324	8 618 178 883	95.49	83.34	94.36	86.70
	GraphChainer							
	minigraph	4.97	12.077	1 415 023 034	17.76	2.51	6.21	3.92
	minichain							
	– – – – – – – – – – – – – – – – – – – – – – – – – – – – – – – – – – –							
	SRFAligner	*3.85	4.817	35 012 334	87.04	75.26	84.27	77.92
	SRFAligner -o 50	6.46	13 767	199 277 440	*97.25	**85.02**	**95.31**	**87.74**
	SRFChainer	4.70	*4.795	*34 135 233	86.95	75.16	84.16	77.83
	SRFChainer -o 50	5.48	5.191	41 039 832	96.47	84.23	95.20	87.59

a Dashed line separates competing tools from our tools. Best performances in each part are highlighted by boldface and overall best performances are marked with “*.”

The tools GraphChainer and minichain took >24 h on chr22-iEFG and were considered to time-out. This high resource usage of minichain and GraphChainer is explained by the high width of chr22-iEFG (see Table 1), as their performance is highly dependent on this parameter. The low accuracy of minigraph and minichain on complex pangenome graphs with short node labels is a known limitation reported at https://github.com/lh3/minigraph#limitations. The comparison for chr22-iEFG is thus not fully fair, as our aligners are tailored to exploit the uniqueness of iEFGs.

We verified the number and quality of srf seeds obtaned by SRFAligner by computing the full set of srf seeds (full node matches) using the single-thread Aho-Corasick automaton implementation daachorse (Kanda *et al.* 2023). We obtained 78 333 317 seeds for chr22-iEFG and extending these seeds yields accuracies of 88.13, 76.14, 88.57, and 80.61 for path accuracy δ=0.1, δ=0.95 and edit distance accuracy $\sigma_{\text{truth}}=0.1$ and $\sigma_{\text{read}}=0.1$, respectively, proving that SRFAligner finds a high-quality subset (22 826 527 full node seeds) of semi-repeat-free seeds retaining most accuracy.

The parameterized modes of SRFAligner and SRFChainer offer the best performance and highest accuracy on the most widely used metric of δ=0.1, with 4.5x speed-up against default GraphAligner on the same input graph and 1.5x speed-up against GraphAligner on vg graphs. Although chaining does not improve accuracy, it reduces the number of final seeds, and hence the overall speed in most cases and the maximum memory consumption of GraphAligner.

## 6 Future work

We remark that srf-based seeds are not only relevant for iEFGs but promising for all pangenome representations, as they characterize the uniqueness of genomic sequences. We plan to study how to project our seeds to other representations.

While our new construction strategy for iEFGs can exploit parallelization, it still requires the whole MSA as input. This could be avoided by first building an MSA of *founder sequences* directly from a reference and variants (Norri *et al.* 2021) and then applying the segmentation algorithm on this much smaller founder MSA. All required components have already been implemented, so our workflow can be adjusted to this approach. The research question to study is how many founder sequences are required to keep the high quality of read alignments, as each original haplotype (row of MSA) is a recombination of founder sequences and a smaller number of founder sequences means increased number of discontinuities.

## Supplementary Material

btaf225_Supplementary_Data

## Data Availability

The data underlying this article are available in Zenodo, at https://doi.org/10.5281/zenodo.14012881
